# Ascorbate-dependent vasopressor synthesis: a rationale for vitamin C administration in severe sepsis and septic shock?

**DOI:** 10.1186/s13054-015-1131-2

**Published:** 2015-11-27

**Authors:** Anitra C. Carr, Geoffrey M. Shaw, Alpha A. Fowler, Ramesh Natarajan

**Affiliations:** Department of Pathology, University of Otago, Christchurch, PO Box 4345, Christchurch, 8140 New Zealand; Department of Intensive Care Medicine, Christchurch Hospital, Private Bag 4710, Christchurch, 8011 New Zealand; Division of Pulmonary Disease and Critical Care Medicine, Department of Internal Medicine, School of Medicine, Virginia Commonwealth University, Box 980050, Richmond, VA 23298 USA

## Abstract

Severe systemic inflammatory response to infection results in severe sepsis and septic shock, which are the leading causes of death in critically ill patients. Septic shock is characterised by refractory hypotension and is typically managed by fluid resuscitation and administration of catecholamine vasopressors such as norepinephrine. Vasopressin can also be administered to raise mean arterial pressure or decrease the norepinephrine dose. Endogenous norepinephrine and vasopressin are synthesised by the copper-containing enzymes dopamine β-hydroxylase and peptidylglycine α-amidating monooxygenase, respectively. Both of these enzymes require ascorbate as a cofactor for optimal activity. Patients with severe sepsis present with hypovitaminosis C, and pre-clinical and clinical studies have indicated that administration of high-dose ascorbate decreases the levels of pro-inflammatory biomarkers, attenuates organ dysfunction and improves haemodynamic parameters. It is conceivable that administration of ascorbate to septic patients with hypovitaminosis C could improve endogenous vasopressor synthesis and thus ameliorate the requirement for exogenously administered vasopressors. Ascorbate-dependent vasopressor synthesis represents a currently underexplored biochemical mechanism by which ascorbate could act as an adjuvant therapy for severe sepsis and septic shock.

## Introduction

Sepsis is a potentially life-threatening systemic inflammatory response to infection which can result in multisystem organ dysfunction (severe sepsis) and refractory hypotension (septic shock) [1]. Sepsis is a complex, heterogeneous condition that can be caused by any infectious organism, with Gram-positive infections often predominating [2]. A majority of septic patients have a pulmonary source of infection, such as pneumonia, which results in acute respiratory distress syndrome [1]. Dysfunction of cardiovascular, hepatic, renal and central nervous systems can also occur in severe sepsis [3]. The incidence of severe sepsis is increasing, due to an increasingly aging population [4], and is currently the leading cause of death in critically ill patients, with mortality rates of 30–50 % [5]. The Surviving Sepsis Campaign has recommended a number of strategies for the management of severe sepsis and septic shock, including fluid resuscitation, infection control, and respiratory and central nervous system support [6] and, despite an increase in incidence, mortality rates appear to be declining [7].

Septic shock is characterised by severe alterations in the cardiovascular system, including ineffective tissue oxygenation, inappropriate peripheral vasodilation, myocardial dysfunction and altered blood flow distribution, resulting in hypotension refractory to intravenous fluid administration [1, 8]. Septic shock is typically managed through the administration of catecholamine vasopressors (dopamine, norepinephrine or epinephrine), with norepinephrine being the preferred vasoconstrictor [9]. Vasopressin is also recommended in the Surviving Sepsis Campaign guidelines to raise mean arterial pressure to target or to decrease the norepinephrine dose [6]. The Vasopressin and Septic Shock Trial (VASST) indicated that vasopressin provided equivalent mortality rates to norepinephrine in patients already receiving vasopressors [10]. Exogenous vasopressor administration to patients with severe sepsis and septic shock, however, can result in adverse side effects such as decreased cardiac output and cardiac arrest, mesenteric ischaemia, skin necrosis, digital ischaemia and hyponatraemia [11].

Recent pre-clinical and clinical studies have indicated a potential role for ascorbate in ameliorating the pathophysiology of sepsis [12–14]. Numerous studies of septic animals administered high-dose ascorbate have shown improved microvascular changes and survival (reviewed in [14]). A number of mechanisms have been proposed for ascorbate’s observed activities in sepsis, but these have primarily focused on its antioxidant and anti-inflammatory functions, including its effects on signal transduction pathways in vascular cells (reviewed in [13]). In contrast, very little attention has been paid to ascorbate’s enzyme cofactor activities, particularly its role in the synthesis of vasopressors [15]. In this review we present the hypothesis that adjuvant ascorbate could support endogenous vasopressor synthesis in severe sepsis and septic shock through acting as an essential cofactor for the metallo-enzymes involved in the biosynthesis of vasoactive catecholamines and vasopressin.

## Ascorbate requirements in sepsis

Ascorbate is an essential micronutrient with numerous important enzyme cofactor functions in the body [15, 16], as well as potent antioxidant properties [17, 18]. Humans have lost the ability to synthesise ascorbate due to mutations in the gene encoding the terminal biosynthetic enzyme, and ascorbate must therefore be obtained regularly through the diet to prevent hypovitaminosis C and the potentially fatal deficiency disease scurvy. Recommended dietary intakes for ascorbate are typically in the range of 75–110 mg/day [19, 20]. Enteral or parenteral nutrition administered to critically ill patients provides ~100 mg/day ascorbate. However, critically ill patients probably require significantly higher intakes of ascorbate [21] due to enhanced metabolic turnover of vitamin C during the severe inflammatory response. It should also be noted that parenteral rather than enteral nutrition may be required for optimal ascorbate status in critically ill patients because intravenously administered ascorbate bypasses the rate-limiting intestinal uptake of orally administered ascorbate [22].

In healthy fasting humans, circulating levels of ascorbate are typically in the range of 50–70 μmol/l, whereas levels <23 μmol/l are considered marginally deficient (or hypovitaminosis C) and levels <11 μmol/l are considered severely deficient and potentially scorbutic [23]. Several studies have shown that ascorbate levels are low in critically ill patients (i.e. <23 μmol/l), including those with acute respiratory infections [24] and sepsis [12, 25], and are particularly low (i.e. <11 μmol/l) in patients who progress to multiple organ failure [26]. In some studies, ascorbate levels remained lower than those in controls, despite the patients receiving 100–500 mg/day ascorbate either enterally or parenterally [27, 28]. Long et al. [27] demonstrated that up to 3000 mg/day ascorbate was required to return plasma levels of critically ill patients to normal (i.e. 68 μmol/l).

Interestingly, in animals that are able to synthesise ascorbate in their livers, studies have shown increased endogenous synthesis of ascorbate when the animals are exposed to stress [29, 30]. We have also observed enhanced mRNA expression of the ascorbate synthesising enzyme gulonolactone oxidase in lipopolysaccharide-treated mice (R. Natarajan, unpublished observations). Enhanced ascorbate synthesis has also recently been shown in mice exposed to tumour burden [31]. These studies suggest an important physiological requirement for ascorbate in conditions of stress and disease burden. Other studies have shown up to an eight-fold enhancement in the synthesis of ascorbate in animals exposed to drugs, including hypnotics (sedatives), analgesics and muscle relaxants [32, 33], probably as a compensatory mechanism for enhanced metabolism of ascorbate following drug administration [33]. Therefore, it is conceivable that patients with severe infection in intensive care may have enhanced ascorbate requirements not only due to the infectious disease process, but also because of the administration of sedatives and other drugs.

## Ascorbate’s role in vasopressor synthesis

Cases of scurvy, the clinical presentation of severe ascorbate deficiency, have been reported in critically ill patients in intensive care (Table 1) [34–36]. One case presented with multiple organ dysfunction and the intravenous administration of ascorbate resulted in improved cardiovascular functioning within 24 h, particularly increased arterial pressure, decreased heart rate, normalised central venous pressure and a decreased need for catecholamines [35]. Another case exhibited typical symptoms of scurvy as well as severe orthostatic hypotension that failed to respond to fluids [36]. The patient’s ascorbate levels were undetectable and the orthostatic hypotension resolved within 24 h of ascorbate replacement. The authors suggested that the pathogenesis of orthostatic hypotension in the setting of scurvy could result from impaired catecholamine synthesis. Vasopressin may also play a role in preventing or minimising orthostatic hypotension [37], indicating a possible link between ascorbate deficiency and vasopressin insufficiency in this case [36]. High-dose intravenous ascorbate administration to severely burned patients has been shown to decrease the resuscitation volume required [38] and also to decrease the need for vasopressors in some patients (Table 1) [39].

**Table 1:** Summary of studies of ascorbate administration with vasopressor-related endpoints

Study type	Study group	Intervention	Findings	Reference
Pre-clinical	24 rats (8/group)	Centrally administered ascorbate (0, 200, 600 nmol)	↑ vasopressin release	[62]
			↑ antidiuresis	
			↑ natriuresis	
Case report	Hospital patient	Intravenous ascorbate (1,500 mg/day)	↑ arterial pressure	[35]
			↓ heart rate	
			Normalised central venous pressure	
			↓ need for catecholamines	
			Improved multiple organ dysfunction	
Case report	Hospital patient	Oral ascorbate (500 mg/day)	Resolution of orthostatic hypotension	[36]
Clinical (retrospective)	33 severely burned patients (16–17/group)	Intravenous ascorbate (0, 66 mg/kg/h)	↓ fluid requirement	[39]
			↓ number of patients requiring vasopressors	
Clinical (randomised, prospective)	37 severely burned patients (17–18/group)	Intravenous ascorbate (0, 66 mg/kg/h)	↓ resuscitation fluid volume	[38]
			↑ diuresis	
			↓ length of mechanical ventilation	
Clinical (phase I randomised controlled trial)	24 severe sepsis patients (8/group)	Intravenous ascorbate (0, 50, 200 mg/kg/24 h)	↓ systemic organ failure	[12]
			↑ systolic blood pressure^a^	
			↑ mean arterial pressure^a^	

^a^Previously unpublished data (R. Natarajan and A. Fowler)

*↑* increase, *↓* decrease

**Fig. 1:**
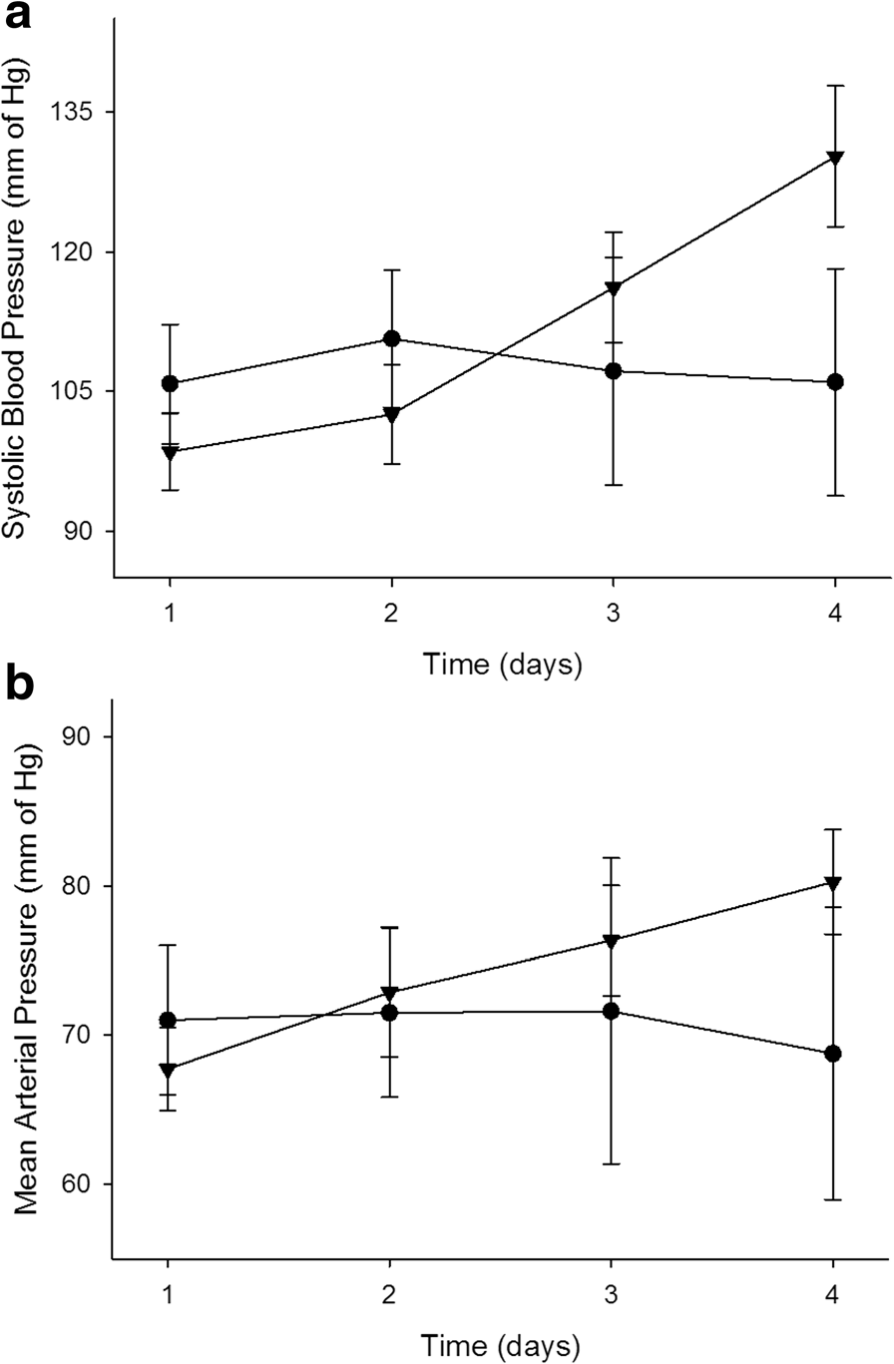
Effect of ascorbate administration on (**a**) systolic blood pressure and (**b**) mean arterial pressure in patients with severe sepsis. Patients were administered placebo (*circle*, 5 % dextrose and water, *n* = 6) or ascorbate (*inverted triangle*, 200 mg/kg/24 h, *n* = 7). The ascorbate dosage was divided into four equal doses and administered over 30 min every 6 h for 96 h in 50 ml of 5 % dextrose and water [12]. Systolic blood pressure and mean arterial pressure were measured at the bedside using an arterial line (radial artery)

The recent phase I randomised controlled trial in 24 patients with severe sepsis who were administered intravenous infusions of ascorbate (0, 50 or 200 mg/kg/24 h) showed prompt reductions in Sequential Organ Failure Assessment scores in those receiving ascorbate [12]. A significant reduction in the pro-inflammatory biomarkers C-reactive protein and procalcitonin was also observed, as well as attenuation of thrombomodulin levels, suggesting amelioration of vascular endothelial injury. No adverse safety events were observed in this study. Previously unpublished data from this trial indicated an increase in systolic blood pressure and mean arterial pressure in the group that received 200 mg/kg/24 h ascorbate (Fig. 1a, b). Although these values did not reach statistical significance, probably due to low patient numbers, this finding lends support to our hypothesis that administration of ascorbate could aid in endogenous vasopressor synthesis.

### Ascorbate and catecholamine synthesis

Catecholamine neurotransmitters and hormones (dopamine, norepinephrine and epinephrine) are synthesised within the sympathetic nervous system and the adrenal medulla (an organ with a very high ability to store vitamin C). Catecholamines increase arterial pressure through binding to and activating α-adrenergic receptors on the smooth muscle cells of the vasculature and can promote increased cardiac contractility and heart rate through binding to β-adrenergic receptors of the cardiac muscle [40]. During sepsis, myocardial depression commonly occurs due to downregulation of β-adrenergic receptors and related cell signalling pathways [41]. Impaired adrenal hormone synthesis has also been observed in critically ill patients and is probably a common complication in severe sepsis [42, 43]. Interestingly, norepinephrine levels are decreased in ascorbate-deficient animal models, particularly in the adrenal glands [44–46]. Ascorbate is also secreted from the adrenal glands as part of the stress response [47], which could conceivably result in adrenal ascorbate depletion under conditions of sustained stress.

**Fig. 2:**
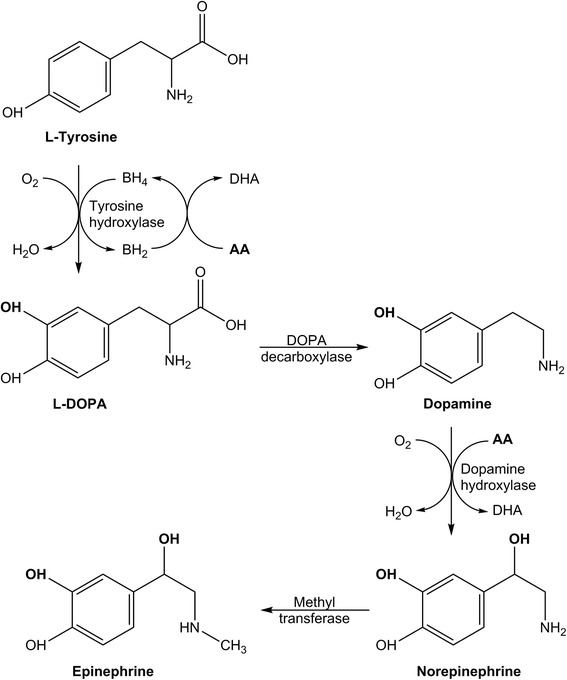
Ascorbate-dependent synthesis of the catecholamine vasopressors dopamine, norepinephrine and epinephrine. Ascorbate acts as a cofactor for the metallo-enzyme dopamine hydroxylase, and also recycles the enzyme cofactor tetrahydrobiopterin. *AA* ascorbic acid, *DHA* dehydroascorbic acid, *BH*_*4*_ tetrahydrobiopterin, *BH*_*2*_ dihydrobiopterin

Ascorbate is required for two enzymatic steps along the catecholamine biosynthetic pathway (Fig. 2). Specifically, ascorbate is a cofactor for the copper-containing enzyme dopamine β-hydroxylase [48, 49]. This enzyme utilises oxygen to introduce a hydroxyl group to dopamine to form norepinephrine (Fig. 2). Epinephrine can then be formed via methyltransferase-catalysed methylation of the amine group of norepinephrine. Recent research indicates that ascorbate may also facilitate the rate-limiting step in the synthesis of dopamine via recycling the enzyme cofactor tetrahydrobiopterin (Fig. 2) [50]. This cofactor is required by the iron-containing enzyme tyrosine hydroxylase which hydroxylates the amino acid l-tyrosine to form the dopamine precursor l-DOPA. Some evidence also suggests that ascorbate enhances the synthesis of tyrosine hydroxylase itself [50]. Ascorbate has also been shown to enhance both α-adrenergic and β-adrenergic receptor activity through binding to the receptor, thereby enhancing its activation by epinephrine [51, 52].

It is noteworthy that tissues where catecholamines are synthesised contain the highest levels of ascorbate in the body (i.e. the brain and adrenal glands) [53], indicating that vitamin C plays a vital role in these organs. Furthermore, animal models of ascorbate deficiency have shown significant retention of the vitamin in the brain during dietary depletion [54–56], supporting the importance of ascorbate in the central nervous system. Thus, appropriate supplementation of ascorbate in sepsis may support endogenous synthesis of vasoactive catecholamines, and possibly also facilitate adrenergic receptor binding.

### Ascorbate and vasopressin synthesis

Vasopressin, also known as arginine vasopressin (AVP) or antidiuretic hormone (ADH), is a peptide hormone which is synthesised in the hypothalamus as a pre-pro-hormone and subsequently stored in the posterior pituitary as the mature hormone [57]. It is secreted in response to decreased blood volume, decreased arterial pressure or increased plasma osmolality, and interacts with specific receptors expressed by vascular smooth muscle cells (AVPR1a) and kidney collecting ducts (AVPR2) to cause vasoconstriction and water retention [11]. The receptor AVPR1b (or AVPR3) is expressed in the anterior pituitary, and stimulation of this receptor by vasopressin enhances release of adrenocorticotrophic hormone (ACTH), thereby acting synergistically with corticotrophin-releasing hormone (CRH), and resulting in stimulation of corticosteroid synthesis in the adrenal cortex in response to stress [11]. Circulating vasopressin levels increase dramatically during the initial phase of septic shock, but this is followed by a significant decline in the latter phase [58, 59]. Patients in late-phase septic shock have significantly lower levels of circulating vasopressin compared with patients in cardiogenic shock, despite similar hypotension [59]. The decline in circulating vasopressin levels after the onset of septic shock is due to depletion of pituitary stores and possibly also impaired vasopressin synthesis [60].

Ascorbate is a cofactor for the copper-containing enzyme peptidylglycine α-amidating monooxygenase (PAM) that is required for the endogenous synthesis of vasopressin [61]. It is of interest to note that the pituitary gland, where the enzyme PAM is abundantly expressed, has the highest levels of ascorbate in the body [53]. Thus, it is conceivable that depletion of the PAM cofactor ascorbate during sepsis may contribute to the observed decrease in vasopressin biosynthesis [58–60]. Support for a connection between ascorbate and vasopressin biosynthesis comes from an animal study whereby centrally administered ascorbate enhanced circulating levels of vasopressin and induced antidiuresis (Table 1) [62].

**Fig. 3:**
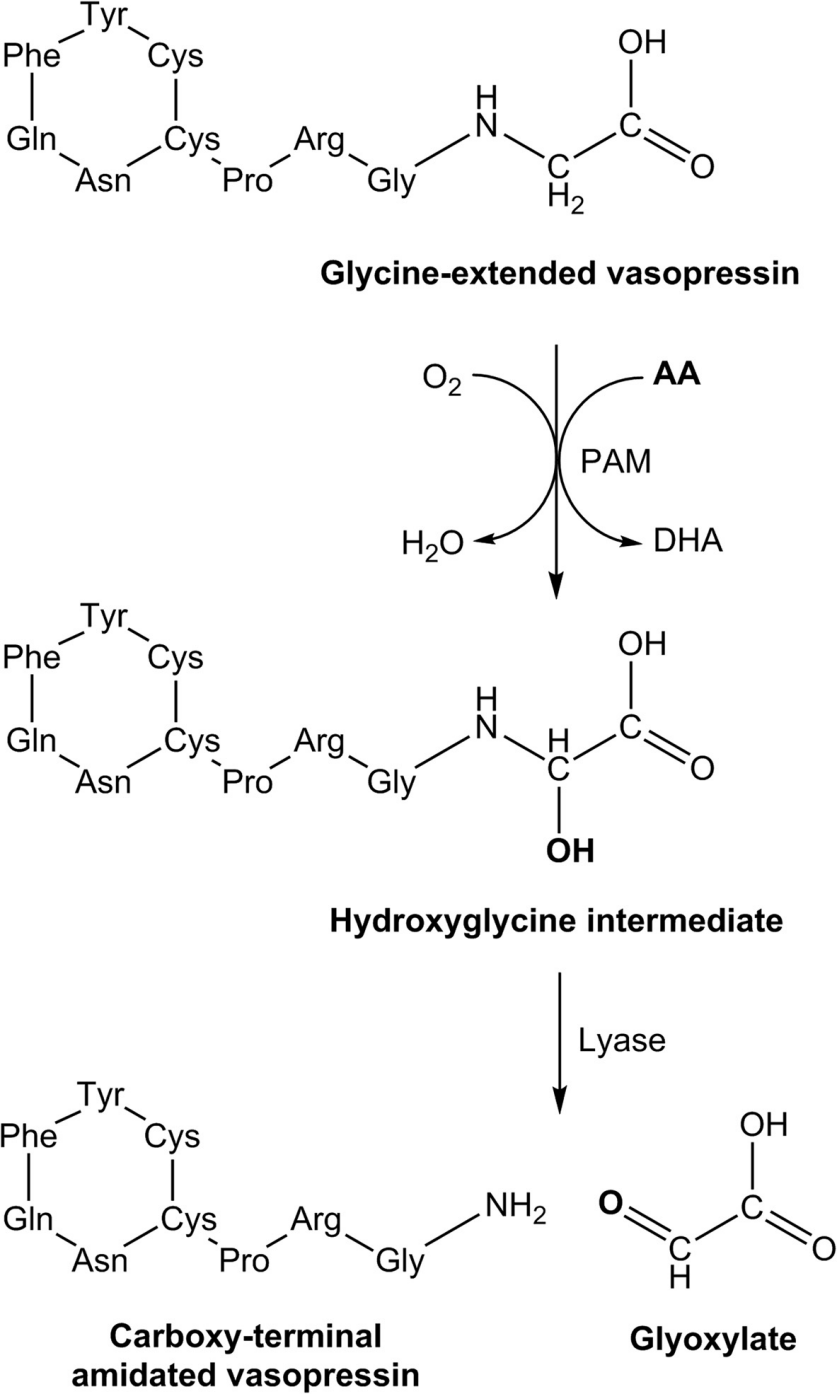
Ascorbate-dependent synthesis of mature carboxy-terminal amidated vasopressin. Ascorbate is a cofactor for the metallo-enzyme peptidylglycine α-amidating monooxygenase (*PAM*). *AA* ascorbic acid, *DHA* dehydroascorbic acid

Vasopressin is synthesised as a pre-pro-hormone which undergoes sequential cleavage steps to produce pro-vasopressin and finally a glycine-extended vasopressin precursor. The carboxy-terminal glycine residue of the vasopressin precursor subsequently undergoes post-translational modification by the ascorbate-dependent enzyme PAM to generate the active carboxy-amidated hormone (Fig. 3). The enzyme comprises two domains: a copper-dependent peptidylglycine α-hydroxylating monooxygenase domain, which converts glycine-extended peptides into a hydroxyglycine intermediate; and a peptidyl α-hydroxyglycine α-amidating lyase domain, which converts the hydroxyglycine intermediate into an amidated product [61]. The carboxy-terminal amine group of amidated peptides is essential for their biological activities [63].

Pro-vasopressin and copeptin, which lack the carboxy-terminal amine present in mature vasopressin, have been proposed as surrogate measures of the mature amidated hormone because of their enhanced half-life in circulation [64, 65]. Pro-vasopressin and copeptin levels have been measured in patients with septic shock and were shown to be significantly associated with mortality, higher levels being observed in non-survivors than in survivors [64, 65]. Circulating copeptin levels are approximately 10-fold higher than those of vasopressin in severe sepsis and septic shock [66]; however, it is not known whether this is simply due to enhanced stability of copeptin in circulation and/or decreased synthesis of mature vasopressin. The assumption that pro-vasopressin/copeptin levels reflect those of active vasopressin in a stoichiometric manner would not hold true if post-translational modification of glycine-extended vasopressin by PAM were affected through limited cofactor availability. In support of this premise, critically ill patients with sepsis and systemic inflammatory response syndrome (SIRS) have a significantly lower ratio of vasopressin to copeptin than patients after cardiac surgery [67]. Thus, pro-vasopressin/copeptin should not be used as a surrogate marker for vasopressin levels in patients with hypovitaminosis C or hypoxia, both of which are common in severe sepsis and septic shock.

## Conclusions

Ascorbate enhances the synthesis of the vasopressors norepinephrine and vasopressin by acting as a cofactor for their respective biosynthetic enzymes. As such, we hypothesise that administration of high-dose ascorbate in conditions of hypovitaminosis C (e.g. severe sepsis and septic shock) may support the endogenous synthesis of these vasoactive compounds and thus ameliorate the need for exogenously administered vasopressors. Ascorbate-dependent vasopressor synthesis represents a plausible physiological mechanism whereby ascorbate could act as an adjuvant therapy for severe sepsis and septic shock. Whether this mechanism translates into improved patient outcomes requires testing in well-designed clinical trials.

## Abbreviations

ACTH: Adrenocorticotrophic hormone
ADH: Antidiuretic hormone
AVP: Arginine vasopressin
CRH: Corticotrophin-releasing hormone
PAM: Peptidylglycine α-amidating monooxygenase
SIRS: Systemic inflammatory response syndrome
VASST: Vasopressin and Septic Shock Trial

## References

[1] Remick DG (2007). Pathophysiology of sepsis. Am J Pathol.

[2] Martin GS, Mannino DM, Eaton S, Moss M (2003). The epidemiology of sepsis in the United States from 1979 through 2000. N Engl J Med.

[3] Vincent JL (2006). Organ dysfunction in patients with severe sepsis. Surg Infect.

[4] Angus DC, Linde-Zwirble WT, Lidicker J, Clermont G, Carcillo J, Pinsky MR (2001). Epidemiology of severe sepsis in the United States: analysis of incidence, outcome, and associated costs of care. Crit Care Med.

[5] Dombrovskiy VY, Martin AA, Sunderram J, Paz HL (2007). Rapid increase in hospitalization and mortality rates for severe sepsis in the United States: a trend analysis from 1993 to 2003. Crit Care Med.

[6] Dellinger RP, Levy MM, Rhodes A, Annane D, Gerlach H, Opal SM, Sevransky JE, Sprung CL, Douglas IS, Jaeschke R, Osborn TM, Nunnally ME, Townsend SR, Reinhart K, Kleinpell RM, Angus DC, Deutschman CS, Machado FR, Rubenfeld GD, Webb SA, Beale RJ, Vincent JL, Moreno R (2013). Surviving sepsis campaign: international guidelines for management of severe sepsis and septic shock: 2012. Crit Care Med.

[7] Lagu T, Rothberg MB, Shieh MS, Pekow PS, Steingrub JS, Lindenauer PK (2012). Hospitalizations, costs, and outcomes of severe sepsis in the United States 2003 to 2007. Crit Care Med.

[8] Angus DC, van der Poll T (2013). Severe sepsis and septic shock. N Engl J Med.

[9] Vasu TS, Cavallazzi R, Hirani A, Kaplan G, Leiby B, Marik PE (2012). Norepinephrine or dopamine for septic shock: systematic review of randomized clinical trials. J Intensive Care Med.

[10] Russell JA, Walley KR, Singer J, Gordon AC, Hebert PC, Cooper DJ, Holmes CL, Mehta S, Granton JT, Storms MM, Cook DJ, Presneill JJ, Ayers D (2008). Vasopressin versus norepinephrine infusion in patients with septic shock. N Engl J Med.

[11] Russell JA (2011). Bench-to-bedside review: vasopressin in the management of septic shock. Crit Care.

[12] Fowler AA, Syed AA, Knowlson S, Sculthorpe R, Farthing D, DeWilde C, Farthing CA, Larus TL, Martin E, Brophy DF, Gupta S, Fisher BJ, Natarajan R (2014). Phase I safety trial of intravenous ascorbic acid in patients with severe sepsis. J Transl Med.

[13] Wilson JX (2013). Evaluation of vitamin C for adjuvant sepsis therapy. Antioxid Redox Signal.

[14] Oudemans-van Straaten HM, Spoelstra-de Man AM, de Waard MC (2014). Vitamin C revisited. Crit Care.

[15] Englard S, Seifter S (1986). The biochemical functions of ascorbic acid. Annu Rev Nutr.

[16] Du J, Cullen JJ, Buettner GR (1826). Ascorbic acid: chemistry, biology and the treatment of cancer. Biochim Biophys Acta.

[17] Carr AC, Frei B (1999). Toward a new recommended dietary allowance for vitamin C based on antioxidant and health effects in humans. Am J Clin Nutr.

[18] Carr A, Frei B (1999). Does vitamin C act as a pro-oxidant under physiological conditions?. Faseb J.

[19] Institute of Medicine Panel on Dietary Antioxidants and Related Compounds (2000). Dietary reference intakes for vitamin C, vitamin E, selenium, and Carotenoids.

[20] New reference values for vitamin C intake. Ann Nutr Metab 2015;67:13–20. 10.1159/000434757 26227083

[21] Berger MM (2009). Vitamin C, requirements in parenteral nutrition. Gastroenterology.

[22] Padayatty SJ, Sun H, Wang Y, Riordan HD, Hewitt SM, Katz A, Wesley RA, Levine M (2004). Vitamin C pharmacokinetics: implications for oral and intravenous use. Ann Intern Med.

[23] Lykkesfeldt J, Poulsen HE (2010). Is vitamin C supplementation beneficial? Lessons learned from randomised controlled trials. Br J Nutr.

[24] Hunt C, Chakravorty NK, Annan G, Habibzadeh N, Schorah CJ (1994). The clinical effects of vitamin C supplementation in elderly hospitalised patients with acute respiratory infections. Int J Vitam Nutr Res.

[25] Schorah CJ, Downing C, Piripitsi A, Gallivan L, Al-Hazaa AH, Sanderson MJ, Bodenham A (1996). Total vitamin C, ascorbic acid, and dehydroascorbic acid concentrations in plasma of critically ill patients. Am J Clin Nutr.

[26] Borrelli E, Roux-Lombard P, Grau GE, Girardin E, Ricou B, Dayer J, Suter PM (1996). Plasma concentrations of cytokines, their soluble receptors, and antioxidant vitamins can predict the development of multiple organ failure in patients at risk. Crit Care Med.

[27] Long CL, Maull KI, Krishnan RS, Laws HL, Geiger JW, Borghesi L, Franks W, Lawson TC, Sauberlich HE (2003). Ascorbic acid dynamics in the seriously ill and injured. J Surg Res.

[28] Metnitz PG, Bartens C, Fischer M, Fridrich P, Steltzer H, Druml W (1999). Antioxidant status in patients with acute respiratory distress syndrome. Intensive Care Med.

[29] Nakano K, Suzuki S (1984). Stress-induced change in tissue levels of ascorbic acid and histamine in rats. J Nutr.

[30] Lahiri S, Lloyd BB (1962). The effect of stress and corticotrophin on the concentrations of vitamin C in blood and tissues of the rat. Biochem J.

[31] Campbell EJ, Vissers MC, Bozonet S, Dyer A, Robinson BA, Dachs GU (2015). Restoring physiological levels of ascorbate slows tumor growth and moderates HIF-1 pathway activity in Gulo(–/–) mice. Cancer Med.

[32] Burns JJ, Mosbach EH, Schulenberg S (1954). Ascorbic acid synthesis in normal and drug-treated rats, studied with L-ascorbic-1-C14 acid. J Biol Chem.

[33] Conney AH, Bray GA, Evans C, Burns JJ (1961). Metabolic interactions between L-ascorbic acid and drugs. Ann N Y Acad Sci.

[34] Holley AD, Osland E, Barnes J, Krishnan A, Fraser JF (2011). Scurvy: historically a plague of the sailor that remains a consideration in the modern intensive care unit. Intern Med J.

[35] Kieffer P, Thannberger P, Wilhelm JM, Kieffer C, Schneider F (2001). Multiple organ dysfunction dramatically improving with the infusion of vitamin C: more support for the persistence of scurvy in our ‘welfare’ society. Intensive Care Med.

[36] Zipursky JS, Alhashemi A, Juurlink D (2014). A rare presentation of an ancient disease: scurvy presenting as orthostatic hypotension. BMJ Case Rep.

[37] Saad CI, Ribeiro AB, Zanella MT, Mulinari RA, Gavras I, Gavras H (1988). The role of vasopressin in blood pressure maintenance in diabetic orthostatic hypotension. Hypertension.

[38] Tanaka H, Matsuda T, Miyagantani Y, Yukioka T, Matsuda H, Shimazaki S (2000). Reduction of resuscitation fluid volumes in severely burned patients using ascorbic acid administration: a randomized, prospective study. Arch Surg.

[39] Kahn SA, Beers RJ, Lentz CW (2011). Resuscitation after severe burn injury using high-dose ascorbic acid: a retrospective review. J Burn Care Res.

[40] De Backer D, Scolletta S (2013). Clinical management of the cardiovascular failure in sepsis. Curr Vasc Pharmacol.

[41] Rudiger A, Singer M (2007). Mechanisms of sepsis-induced cardiac dysfunction. Crit Care Med.

[42] Nieboer P, van der Werf TS, Beentjes JA, Tulleken JE, Zijlstra JG, Ligtenberg JJ (2000). Catecholamine dependency in a polytrauma patient: relative adrenal insufficiency?. Intensive Care Med.

[43] Duggan M, Browne I, Flynn C (1998). Adrenal failure in the critically ill. Br J Anaesth.

[44] Hoehn SK, Kanfer JN (1980). Effects of chronic ascorbic acid deficiency on guinea pig lysosomal hydrolase activities. J Nutr.

[45] Deana R, Bharaj BS, Verjee ZH, Galzigna L (1975). Changes relevant to catecholamine metabolism in liver and brain of ascorbic acid deficient guinea-pigs. Int J Vitam Nutr Res.

[46] Bornstein SR, Yoshida-Hiroi M, Sotiriou S, Levine M, Hartwig HG, Nussbaum RL, Eisenhofer G (2003). Impaired adrenal catecholamine system function in mice with deficiency of the ascorbic acid transporter (SVCT2). FASEB J.

[47] Padayatty SJ, Doppman JL, Chang R, Wang Y, Gill J, Papanicolaou DA, Levine M (2007). Human adrenal glands secrete vitamin C in response to adrenocorticotrophic hormone. Am J Clin Nutr.

[48] Levine M (1986). Ascorbic acid specifically enhances dopamine beta-monooxygenase activity in resting and stimulated chromaffin cells. J Biol Chem.

[49] May JM, Qu ZC, Nazarewicz R, Dikalov S (2013). Ascorbic acid efficiently enhances neuronal synthesis of norepinephrine from dopamine. Brain Res Bull.

[50] May JM, Qu ZC, Meredith ME (2012). Mechanisms of ascorbic acid stimulation of norepinephrine synthesis in neuronal cells. Biochem Biophys Res Commun.

[51] Dillon PF, Root-Bernstein RS, Lieder CM (2004). Antioxidant-independent ascorbate enhancement of catecholamine-induced contractions of vascular smooth muscle. Am J Physiol Heart Circ Physiol.

[52] Dillon PF, Root-Bernstein R, Robinson NE, Abraham WM, Berney C (2010). Receptor-mediated enhancement of beta adrenergic drug activity by ascorbate in vitro and in vivo. PLoS One.

[53] Hornig D (1975). Distribution of ascorbic acid, metabolites and analogues in man and animals. Ann N Y Acad Sci.

[54] Vissers MCM, Bozonet SM, Pearson JF, Braithwaite LJ (2011). Dietary ascorbate affects steady state tissue levels in vitamin C-deficient mice: tissue deficiency after sub-optimal intake and superior bioavailability from a food source (kiwifruit). Am J Clin Nutr.

[55] Hughes RE, Hurley RJ, Jones PR (1971). The retention of ascorbic acid by guinea-pig tissues. Br J Nutr.

[56] Hasselholt S, Tveden-Nyborg P, Lykkesfeldt J (2015). Distribution of vitamin C is tissue specific with early saturation of the brain and adrenal glands following differential oral dose regimens in guinea pigs. Br J Nutr.

[57] Treschan TA, Peters J (2006). The vasopressin system: physiology and clinical strategies. Anesthesiology.

[58] Landry DW, Levin HR, Gallant EM, Ashton RC, Seo S, D'Alessandro D, Oz MC, Oliver JA (1997). Vasopressin deficiency contributes to the vasodilation of septic shock. Circulation.

[59] Sharshar T, Blanchard A, Paillard M, Raphael JC, Gajdos P, Annane D (2003). Circulating vasopressin levels in septic shock. Crit Care Med.

[60] Sharshar T, Carlier R, Blanchard A, Feydy A, Gray F, Paillard M, Raphael JC, Gajdos P, Annane D (2002). Depletion of neurohypophyseal content of vasopressin in septic shock. Crit Care Med.

[61] Prigge ST, Mains RE, Eipper BA, Amzel LM (2000). New insights into copper monooxygenases and peptide amidation: structure, mechanism and function. Cell Mol Life Sci.

[62] Giusti-Paiva A, Domingues VG (2010). Centrally administered ascorbic acid induces antidiuresis, natriuresis and neurohypophyseal hormone release in rats. Neuro Endocrinol Lett.

[63] Merkler DJ (1994). C-terminal amidated peptides: production by the in vitro enzymatic amidation of glycine-extended peptides and the importance of the amide to bioactivity. Enzyme Microb Technol.

[64] Guignant C, Voirin N, Venet F, Poitevin F, Malcus C, Bohe J, Lepape A, Monneret G (2009). Assessment of pro-vasopressin and pro-adrenomedullin as predictors of 28-day mortality in septic shock patients. Intensive Care Med.

[65] Morgenthaler NG, Muller B, Struck J, Bergmann A, Redl H, Christ-Crain M (2007). Copeptin, a stable peptide of the arginine vasopressin precursor, is elevated in hemorrhagic and septic shock. Shock.

[66] Jochberger S, Dorler J, Luckner G, Mayr VD, Wenzel V, Ulmer H, Morgenthaler NG, Hasibeder WR, Dunser MW (2009). The vasopressin and copeptin response to infection, severe sepsis, and septic shock. Crit Care Med.

[67] Jochberger S, Morgenthaler NG, Mayr VD, Luckner G, Wenzel V, Ulmer H, Schwarz S, Hasibeder WR, Friesenecker BE, Dunser MW (2006). Copeptin and arginine vasopressin concentrations in critically ill patients. J Clin Endocrinol Metab.

